# Evaluating Adult Cystic Fibrosis Care in BC: Disparities in Access to a Multidisciplinary Treatment Centre

**DOI:** 10.1155/2016/8901756

**Published:** 2016-02-29

**Authors:** James M. Roberts, Pearce G. Wilcox, Bradley S. Quon

**Affiliations:** ^1^MD Undergraduate Program, University of British Columbia, Vancouver, BC, Canada; ^2^Centre for Heart Lung Innovation, University of British Columbia and St. Paul's Hospital, Vancouver, BC, Canada; ^3^Division of Respiratory Medicine, Department of Medicine, University of British Columbia, 1081 Burrard Street, 8B Providence Wing, Vancouver, BC, Canada V6Z 1Y6

## Abstract

*Background*. Cystic fibrosis (CF) care that is delivered through dedicated, multidisciplinary CF clinics is believed to be partly responsible for improvements in the length and quality of life of persons with CF. We hypothesized patients living farthest from a CF clinic would be seen less frequently than recommended, which would result in reduced access to guideline-recommended care and poorer health outcomes.* Methods*. We performed a retrospective chart review of 168 patients who accessed CF care primarily through the St. Paul's Hospital Adult CF Clinic. Subjects were stratified into four geographical groups according to the estimated one-way travel time by automobile from their home address to the clinic (<45 mins, 45–150 mins, 150–360 mins, and >360 mins).* Results*. There were no significant differences in pulmonary function, nutritional status, CF-related complications, or access to guideline-recommended CF pulmonary therapies between the four groups. Compared to the reference (<45 mins) group, subjects in the two farthest groups (>150 mins) were less likely to be seen in the clinic quarterly as recommended by current CF care guidelines (*p* = 0.002). Those in the farthest group (>360 mins) were at risk for more rapid decline in lung function compared to the reference group (FEV1% predicted annual change: −3.1%/year [95% CI −5.1 to −1.1] versus −0.9%/year [95% CI −1.6 to 0.1], resp., *p* = 0.04).* Conclusions*. Access to CF care is a challenge for individuals who live outside Metro Vancouver and has health policy implications. Further initiatives should be undertaken to ensure equitable care for people living with CF.

## 1. Introduction

Cystic fibrosis (CF) is a common fatal genetic disorder affecting 1 of 3,600 Caucasians born in Canada [1]. It affects multiple organ systems, most notably the lungs, pancreas, sinuses, and gastrointestinal tract. Due to the complex nature of CF care, current guidelines recommend that individuals with CF be seen at a dedicated CF clinic on a quarterly basis at a minimum [2, 3]. During clinic encounters, a multidisciplinary team of doctors (including respirologists and psychiatrists), nurses, dieticians, social workers, pharmacists, and physiotherapists assess each patient. Today in Canada, the median age of predicted survival stands at 50.9 years, more than twice the median age of predicted survival in 1974, and 59.8% of all individuals living with CF are adults above the age of eighteen [4]. Multidisciplinary care delivered through CF clinics on a routine, frequent basis is believed to be responsible, at least in part, for the improvements in CF clinical outcomes and survival observed over the past four decades [5–7].

Despite advances in CF care, it remains challenging for patients who live far from CF clinics to be seen on a quarterly basis as recommended. This is especially the case in British Columbia, where the CF population is dispersed over a large geographic area and the only two adult CF clinics are concentrated in the southwest corner of the province. The two BC adult CF clinics, located in Vancouver and Victoria, provide specialized care for the entire population of nearly three hundred adults living with CF. The Victoria Adult CF Clinic primarily serves individuals with CF living on Vancouver Island, while the St. Paul's Hospital (SPH) Adult CF Clinic in downtown Vancouver primarily serves the Metro Vancouver area and much of the rest of the mainland.

**Figure 1:**
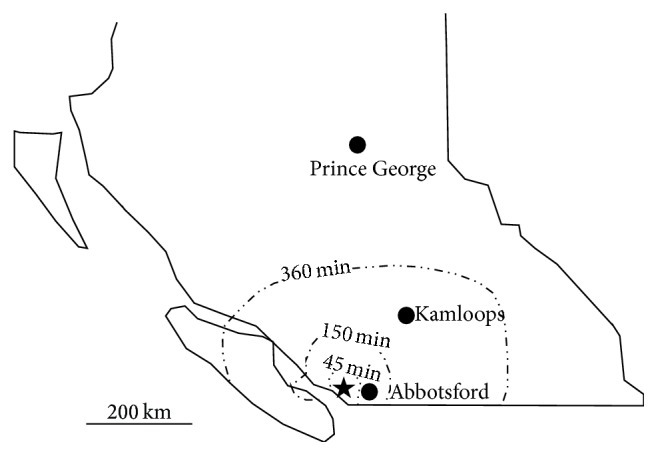
One-way travel time by automobile isochrones to St. Paul's Hospital in Vancouver (star) along major roadways. Points on the map where major routes do not exist were interpolated. Satellite clinic locations are denoted by shaded circles.

In an attempt to address concerns about reduced access to care for patients living outside of the Metro Vancouver region, the St. Paul's Hospital Adult CF Clinic hosts satellite outreach clinics biannually in Kamloops and Prince George and annually in Abbotsford (Figure 1). Despite these outreach efforts, many geographically isolated patients still find it challenging to be seen quarterly. In a recent clinic survey of 132 St. Paul's Hospital Adult CF Clinic patients, 25.0% described their travel time to get to clinic appointments as either “fair” or “poor.” A number of respondents expressed that it was inconvenient and time consuming to access clinic services in Vancouver on a routine basis.

We examined the demographics, therapies, and health outcomes of individuals who were registered with the St. Paul's Hospital Adult CF Clinic. Because CF care for these individuals is directed though St. Paul's Hospital in Vancouver, we hypothesized that clinic patients living farthest from the Metro Vancouver area would be seen less frequently than recommended, which would result in reduced access to CF guideline-recommended care and poorer health outcomes. To our knowledge, no prior studies have investigated the potential impacts of travel time to a multidisciplinary CF clinic.

## 2. Methods

### 2.1. Study Design

We performed a retrospective chart review of patients registered with the St. Paul's Hospital Adult CF Clinic for the period 1st April 2013 to 31st March 2014 (the “study period”). Excluded from the study were individuals whose primary CF care was provided by a different clinic (e.g., transplant clinics and other CF clinics), individuals without a confirmed diagnosis of cystic fibrosis based on standard criteria [8], individuals who were either transplanted or deceased during the study period, and individuals who joined the clinic after the start of the study period.

### 2.2. Data Source

We reviewed paper charts and electronic health records of subjects who were on the St. Paul's Hospital Adult CF Clinic registry and who met the inclusion criteria.

Demographic data for each subject was recorded, including birth year, age at diagnosis, postal code, genotype, and sweat chloride test results. CFTR genotype was documented in terms of F508del-CFTR mutation homozygous or other.

We documented the incidence of five common CF-related complications in study subjects: pancreatic insufficiency, CF-related diabetes (CFRD), chronic sinusitis, low bone mineral density, and depression and/or anxiety. For the purpose of this study, subjects were judged to be pancreatic insufficient if they were prescribed pancreatic enzyme replacement therapy or otherwise had reported symptoms of pancreatic insufficiency (e.g., steatorrhea). A diagnosis of CFRD was established on the basis of a 2-hour blood glucose greater than 11 mmol/L following administration of a 75 g oral glucose tolerance test (OGTT), with or without fasting hyperglycemia. The criterion for low bone mineral density included a diagnosis of osteopenia or osteoporosis, that is,* T*-score of less than −1.0. Depression, anxiety, and chronic sinusitis were clinical diagnoses. Many individuals with CF may present with some degree of depression, anxiety, or sinusitis; these complications were only documented if there was strong evidence for their diagnosis in the electronic health record. This evidence included specialist consultations or recent documentation of medications being prescribed to treat these conditions.

We recorded longitudinal clinical outcome measurements at each routine clinic visit, including pulmonary function and nutritional status, characterized by forced expiratory volume in 1 second (FEV1) (% predicted) and body mass index (BMI), respectively. Pulmonary function and BMI measurements from hospitalization records were not included. The FEV1% predicted and BMI measurements were averaged to generate an annual value for each subject.

The number of routine clinic visits attended, surveillance tests performed, and screening tests performed for each subject during the study period were also documented. In this study, routine clinic visits and routine surveillance tests were defined as those visits or tests not associated with a subsequent hospitalization and also included satellite outreach clinics.

For the purposes of this study, surveillance testing included sputum cultures (routine bacterial culture to detect CF pathogens and acid-fast bacillus culture for nontuberculous mycobacteria) and screening tests included attainment of CF-specific blood work on an annual basis (e.g., serum assays for vitamins A, D, and E) and bone mineral density scans.

Finally, we recorded information regarding access to Grades A and B guideline-recommended pulmonary therapies, that is, those with moderate to high certainty and estimate of net benefit based on lung disease severity and sputum microbiology [9]. In particular, we recorded access to nebulized mucolytics (i.e., dornase alfa) and hyperosmolar agents (i.e., hypertonic saline), three-time weekly or daily oral azithromycin, and cyclical inhaled antibiotics. Access to a therapy was defined as prescription of these therapies as documented in the electronic health records. Ivacaftor, a new* CFTR* potentiator, is included as a Grade A recommendation in pulmonary therapy guidelines. Its use, however, was not recorded in our study due to its relatively recent introduction and the small number of individuals for whom it is presently indicated.

### 2.3. Travel Time Groups

The driving time and distance to St. Paul's Hospital from the subject's home were estimated using Google Maps' “Directions” feature using postal code data, and the shortest driving route was used. The subjects were then assigned to one of four groups based on their estimated one-way travel time by automobile to St. Paul's Hospital in Vancouver: 0–45 minutes, 45–150 minutes, 150–360 minutes, and more than 360 minutes (Figure 1). We selected these travel time groups so as to provide geographic relevance to our results. The first group included Vancouver and the immediate surrounding area, the second group included primarily the Fraser Valley, the third group included Vancouver Island and the Southern Interior, and the fourth group included Northern BC and the Kootenays. Since it was not possible to comprehensively capture the choice of transportation for all visits (e.g., air travel and public transportation), we elected using the subject's one-way travel time by automobile as this metric reflects the effort and resources typically required to access clinic services.

### 2.4. Cross-Sectional and Longitudinal Analyses

Cross-sectional outcomes were analyzed during the study period (defined above), including frequency of routine clinic visits, screening and surveillance testing, rates of CF complications, and rates of access to guideline-recommended pulmonary therapies.

We analyzed data from the subset of individuals who were followed up for three consecutive years from 1st April 2011 until 31st March 2014 (i.e., the study period and two full years preceding) to determine the annual rate of change in FEV1 (% predicted) and BMI (Table 3). This longitudinal analysis was performed to determine whether a group was at a greater risk of lung function decline or nutritional status deterioration, as such differences might be overlooked in cross-sectional analysis.

### 2.5. Statistical Analysis

Groups were compared using one-way ANOVA, repeated-measures ANOVA, or the Kruskal-Wallis test (as appropriate) for continuous variables and Fisher's exact test for dichotomous variables. Rates of change in FEV1% predicted and BMI were modeled using multilevel mixed-effects linear regression. Statistical analyses were performed using MATLAB R2013a (MathWorks, Natick, MA) and STATA 12.0 (Statacorp, College Station, TX).

### 2.6. Ethics Approval

The University of British Columbia Providence Health Care Research Institute Research Ethics Board (UBC-PHC REB number H14-01051) reviewed and accepted the research protocol for this study. Individual patient consent was not obtained or required by the UBC-PHC REB.

## 3. Results

### 3.1. Study Cohort Selection and Travel Time to CF Clinic

**Figure 2:**
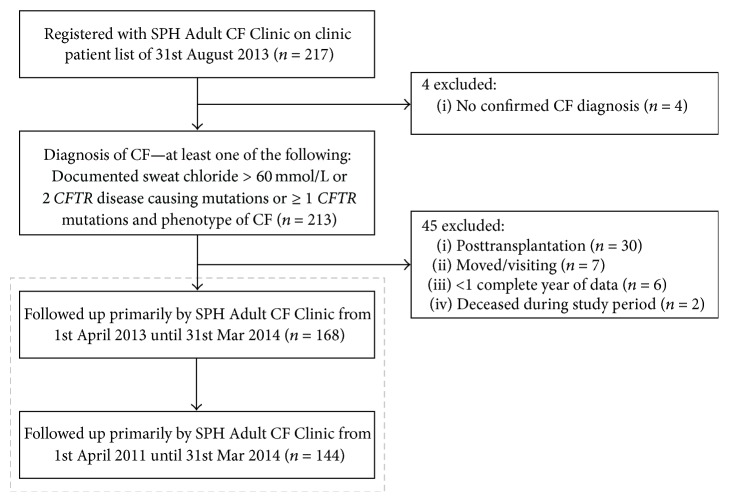
Study inclusion and exclusion criteria.

Of the 213 potential study subjects registered with the CF clinic during the study period who met the diagnostic criteria for cystic fibrosis, 45 (21.1%) were excluded; two-thirds of these exclusions were due to posttransplantation status (30 of 45, 66.7%) (Figure 2). A total of 168 adults with CF followed primarily by the St. Paul's Hospital Adult CF Clinic during the study period were therefore included. The number of subjects in each group was as follows: 85 (<45 mins), 28 (45–150 mins), 42 (150–360 mins), and 13 (>360 mins). The median one-way travel time by automobile for subjects in each group were 27 mins, 56 mins, 324 mins, and 576 mins for the four travel time groups, respectively.

### 3.2. Baseline Characteristics, Disease Severity, and CF Complications

The study population consisted of 97 men (58%) and 71 women (42%), with a mean age of 34.9 (standard deviation 12.5) years. Seventy-one subjects (42%) were homozygous for the F508del-CFTR mutation. Across all subjects, the median age of diagnosis was 3.5 (IQR 15.0) years.

The mean age, sex, age at diagnosis, F508del genotype status, and rates of five of the commonest complications of CF did not differ significantly between groups (Table 1). The mean FEV1% predicted, mean BMI, and proportion of subjects chronically infected with* Pseudomonas aeruginosa*, as defined by the presence of* P. aeruginosa* isolates in ≥50% of routine sputum cultures during the study period [10], also did not vary significantly between groups (Table 1).

### 3.3. Access to Clinic, Screening and Surveillance Tests, and Guideline-Recommended Pulmonary Therapies

Individuals who lived in the two groups farthest from St. Paul's Hospital (i.e., greater than 2.5 hours one-way travel time by automobile) were less likely to meet the guideline-recommended routine quarterly clinic visits and were less likely to provide the recommended four routine sputum samples annually (Table 2). All groups had similar proportions of acid-fast bacillus sputum cultures performed, CF-related annual bloodwork recorded, and bone mineral density scans performed at least once during the study period. Across the four groups, access to six selected Grades A and B recommended that CF pulmonary therapies were also not significantly different (Table 2).

### 3.4. Rates of Change in Pulmonary Function and Nutritional Status

Of the 168 subjects in the cross-sectional group, 144 had three full years of study data and were included in the longitudinal analysis to examine changes in lung function and nutritional status over time. Individuals in the farthest group (>360 mins one-way travel time by automobile) demonstrated a greater absolute rate of decline in lung function compared to the group closest to St. Paul's Hospital (Table 3). Individuals in the third travel time group (150–360 mins one-way travel time by automobile) on average had a mild increase in BMI over the extended study period (Table 3).

## 4. Discussion

The situation of a CF clinic located in a large urban centre and servicing an entire province or state is not unique to British Columbia, and access to cystic fibrosis care remains difficult for many people with the disease in other parts of Canada and abroad. Half of all individuals with CF receiving care at the St. Paul's Hospital Adult CF Clinic had a one-way travel time by automobile of more than 45 minutes, and one-third had a one-way travel time by automobile of more than 2.5 hours. We found that travel time is a substantial barrier for access to routine multidisciplinary CF care, and our work builds on prior studies that have examined the relationship between travel time to a specialized centre and access to care and health outcomes [11, 12].

While the pulmonary function, nutritional status, incidence of CF complications, frequency of bone mineral density testing, frequency of CF-specific bloodwork, and access to CF guideline-recommended therapies did not vary widely across the province, a travel time by automobile of more than 2.5 hours to the clinic was associated with fewer routine clinic visits and fewer routine surveillance sputum cultures analyzed. Routine quarterly clinic visits are particularly important to detect early changes in clinical status, to educate patients on nonpharmaceutical repeated interventions (e.g., chest physiotherapy and dietary regimens), and to ensure surveillance of sputum microbiology. Earlier detection of organisms in the lower respiratory tract can increase the chance of a successful eradication attempt [13]; therefore, it is essential to routinely obtain sputum cultures. Establishment of chronic airway infection by organisms such as* P. aeruginosa* results in worse health outcomes including accelerated decline in lung function and increased mortality [14, 15]. While there were no cross-sectional differences in lung function based on travel time group, a travel time of more than six hours by automobile in each direction was associated with an accelerated rate of lung function decline over the 3-year observational period.

While the proportion of patients with routine quarterly visits from the groups closest to SPH was also less than ideal at just over 50%, patient choice and medical need also influence the frequency of clinic visits. For example, individuals with CF who are diagnosed as adults, particularly those with congenital bilateral absence of the vas deferens, are typically seen twice annually since many of these individuals have milder manifestations of CF. It should also be noted that while there was no difference in the rates of adherence to routine screening tests (e.g., annual blood work and bone density measurement) by travel time group, rates overall were low and suggest room for improvement. The rates of bone mineral density testing were particularly low but these were based on cross-sectional annual measurement and this test is not typically recommended annually [2].

While it is reassuring that CF outcomes are not markedly disparate across the province, this study has important health policy implications. The fewer routine clinic visits for patients living farthest from Vancouver demonstrate that access to CF care remains an issue despite the availability of a British Columbia medical grant (funded by the British Columbia Ministry of Health and administered by CF Canada) that reimburses travel to Vancouver for clinic visits. This suggests there is a role for further outreach to individuals who live outside the Metro Vancouver area. Outreach initiatives may include additional satellite clinics or telehealth access for those who cannot be seen quarterly at the Vancouver CF clinic. Satellite clinics already take place in Kamloops, Prince George, and Abbotsford but appear to be inadequate to meet the growing needs of a geographically dispersed patient population. We are currently in the process of piloting a telemedicine program. A recent systematic review of the literature evaluating telehealth in CF was inconclusive in terms of benefit due to the limited number of published studies but was identified as a promising area for future investigation [16]. Additionally, a shared model of care may be developed whereby increased collaboration with the patient's family doctor and local specialists may help to ensure that at least routine sputum cultures and lung function tests are being performed quarterly for all patients. By increasing the frequency of contact with the clinic and ensuring sputum is cultured routinely, earlier interventions can be sought and disease progress may be slowed for those who experience difficulties accessing clinic services. Home spirometry and electronic symptom diaries might also enhance disease monitoring for patients remote from pulmonary function laboratories [17].

There are several potential limitations to this study. We recognize that some of the individuals in this study may routinely consult local respirology specialists; there was, however, no systematic way to comprehensively document these visits. Indeed, the purpose of this study was to assess access to a multidisciplinary CF clinic, which is recommended by CF care guidelines [2]. Furthermore, we recognize that as an individual's condition deteriorates or as complications accumulate, they may elect to move closer to Vancouver to increase their access to a potential transplant or increase access to additional specialist care. Given the cross-sectional study design, it was not possible to evaluate the impact of this type of migration, which may reduce differences in outcomes and bias the study results towards the null hypothesis. We found no significant differences in lung function between groups when our data was analyzed cross-sectionally but a faster rate of FEV1 decline in the most distant group was evident when this outcome was examined longitudinally.

For this study, we estimated travel time based on the trip time by automobile. We felt this reflects the effort and resources typically required to access CF clinic services but we could not confirm how patients actually travelled to their appointments.

This study did not include patients who had undergone lung transplantation, who are typically followed up by transplantation clinics and who may access CF clinic services on a reduced basis. It is important that this population maintains routine contact with the CF clinic in order to manage their extrapulmonary complications of CF and receive screening tests (e.g., diabetes monitoring and colonoscopy as indicated for colon cancer screening), which may otherwise be overlooked.

Another important outcome in CF is pulmonary exacerbations, but this was not included in our study as there were no reliable means to identify all treated exacerbations around the province without access to provincial administrative healthcare data, which could be the focus of a future study. A previous study, however, performed by Stephenson et al. in Ontario did not notice any differences in hospitalization rates based on socioeconomic status [12]. Exacerbation rates are difficult to interpret when evaluating access to care and socioeconomic status as frequency of visits (i.e., access to care itself) can influence both the identification and treatment of these events. Nevertheless, reduced access to care could lead to delays in initiating exacerbation treatment with the potential for reduced recovery of lung function and should be explored in future studies.

In conclusion, the increased quantity and quality of life of individuals with CF over the past three decades have been significant despite an ongoing search for a cure for this disease. Integrated CF clinics have played a large role in the advances associated with decreased CF mortality and morbidity. It is essential for individuals with CF to remain connected with a dedicated CF clinic at all stages of their disease. Despite the potential limitations of this study, the findings may be generalizable to a large number of CF patients and care providers in other parts of Canada and abroad. While at present some challenges to accessing routine multidisciplinary CF care exist, we have shown that individuals with CF in British Columbia continue to experience similar health outcomes regardless of their location of residence. The approach of a single clinic with an array of multidisciplinary staff, coupled with frequent outreach initiatives, including satellite clinics, telehealth contact, family physician, and local specialist involvement, may be applicable to other “orphan” diseases for which comprehensive multidisciplinary outpatient care might not otherwise be available.

## Figures and Tables

**Table 1:** Baseline characteristics, CF complications, lung function, and nutritional status by travel time.

	One-way travel time by automobile to SPH, min				*p* value
	<45 (*n* = 85)	45–150 (*n* = 28)	150–360 (*n* = 42)	>360 (*n* = 13)	
Baseline characteristics					
Mean age, yr (SD)	34 (11)	38 (15)	36 (14)	32 (10)	0.4
Male sex, *n* (%)	52 (61%)	18 (64%)	21 (50%)	6 (46%)	0.4
Median age at diagnosis, yr (IQR)	5.5 (17.5)	5.5 (19)	2 (11)	1.5 (6)	0.3
F508del-CFTR homozygous, *n* (%)	36 (42%)	9 (32%)	20 (48%)	6 (46%)	0.6
Chronic PsA+, *n* (%)	33 (41%)	14 (54%)	18 (50%)	3 (27%)	0.4
CF complications					
Pancreatic insufficiency, *n* (%)	57 (68%)	20 (71%)	35 (83%)	9 (69%)	0.2
CFRD, *n* (%)	26 (31%)	6 (21%)	11 (27%)	2 (17%)	0.7
Chronic sinusitis, *n* (%)	49 (59%)	16 (57%)	27 (64%)	5 (42%)	0.6
Low BMD, *n* (%)	29 (35%)	11 (39%)	14 (34%)	3 (25%)	0.8
Depression and/or anxiety, *n* (%)	32 (38%)	8 (29%)	7 (17%)	2 (15%)	0.1
Lung function and nutritional status					
FEV1% predicted (SD)	74 (27)	67 (28)	75 (26)	74 (23)	0.6
BMI, kg/m^2^ (SD)	23.7 (4.1)	22.5 (3.9)	23.5 (3.3)	23.2 (1.8)	0.5

Mean (standard deviation), median (intraquartile range), or *n* (%).

SD: standard deviation; BMD: bone mineral density; FEV1: forced expiratory volume in 1 second; BMI: body mass index; PsA+: *P. aeruginosa* isolates in >50% of annual sputum cultures; IQR: intraquartile range; CFRD: CF-related diabetes.

**Table 2:** Access to clinic, surveillance and screening tests, and guideline-recommended treatments by travel time.

	One-way travel time by automobile to SPH, min				*p* value
	<45 (*n* = 85)	45–150 (*n* = 28)	150–360 (*n* = 42)	>360 (*n* = 13)	
*Routine clinic visits*					
Routine clinic visits, mean (SD)	3.8 (2.1)	3.6 (1.9)	2.2 (1.5)^*∗*^	2 (1.5)^*∗*^	<0.001
≥4 routine clinic visits, *n* (%)	45 (53%)	15 (54%)	10 (24%)^*∗*^	2 (15%)^*∗*^	0.002
*Routine surveillance and screening tests*					
Routine sputum cultures, mean (SD)	3.6 (1.9)	3.4 (1.7)	2.4 (1.8)^*∗*^	2.5 (1.9)	0.004
≥4 routine sputum cultures, *n* (%)	47 (55%)	14 (50%)	12 (29%)^*∗*^	4 (31%)	0.023
Bone density measurement, *n* (%)	19 (22%)	11 (39%)	7 (17%)	2 (15%)	0.2
Annual CF bloodwork, *n* (%)	49 (58%)	17 (61%)	25 (60%)	7 (54%)	1.0
≥1 NTM culture, *n* (%)	71 (84%)	24 (86%)	29 (69%)	10 (77%)	0.2
*Access to guideline-recommended pulmonary therapies*					
Grade A: inhaled antibiotics^a^, PsA+ and severe^b^ or moderate^c^ lung disease, *n* (%)	17 (89%)	11 (92%)	8 (80%)	2 (100%)	0.8
Grade A: dornase alfa, severe or moderate lung disease, *n* (%)	19 (59%)	12 (80%)	15 (88%)	3 (60%)	0.1
Grade B: inhaled antibiotics, PsA+ and mild^d^ lung disease, *n* (%)	5 (42%)	1 (50%)	3 (50%)	1 (100%)	0.9
Grade B: dornase alfa, mild lung disease or normal lung function, *n* (%)	18 (38%)	2 (18%)	6 (32%)	2 (67%)	0.7
Grade B: azithromycin, PsA+ and negative NTM culture, *n* (%)	22 (73%)	11 (85%)	13 (81%)	2 (100%)	0.5
Grade B: hypertonic saline, any disease, *n* (%)	35 (41%)	8 (29%)	11 (26%)	3 (23%)	0.3

Mean (standard deviation), or *n* (%).

NTM: nontuberculous mycobacteria; PsA+: *P. aeruginosa* isolates in ≥ 50% of annual sputum cultures.

^a^Inhaled antibiotics included tobramycin (powdered or nebulized) and aztreonam.

^b^Severe lung disease: FEV1% predicted < 40%.

^c^Moderate lung disease: FEV1% predicted 40–69%.

^d^Mild lung disease: FEV1% predicted 70–90%.

*∗* = Statistically significant difference compared to reference group (one-way driving travel time < 45 minutes).

**Table 3:** Longitudinal analysis of rates of absolute change in FEV1% predicted and BMI by travel time.

Absolute change in	One-way travel time by automobile to SPH, min			
	<45 (*n* = 69)	45–150 (*n* = 25)	150–360 (*n* = 39)	>360 (*n* = 11)
FEV1 % predicted/year	−0.9 [−1.6, 0.1]	−0.5 [−1.8, 0.8]	0.1 [−1.0, 1.2]	−3.1^*∗*^ [−5.1, −1.1]
BMI (kg/m^2^)/year	0.0 [−0.1, 0.1]	0.1 [−0.2, 0.3]	0.3^£^ [0.1, 0.5]	−0.3 [−0.6, 0.1]

Mean [95% confidence interval].

FEV1: forced expiratory volume in 1 second; BMI: body mass index.

^*∗*^ *p* = 0.04 in comparison to reference group (one-way driving travel time < 45 mins).

^£^ *p* = 0.01 in comparison to reference group (one-way driving travel time < 45 mins).

The remainder of the comparisons was not statistically significant.
